# Use of Imaging to Optimise Prostate Cancer Tumour Volume Assessment for Focal Therapy Planning

**DOI:** 10.1007/s11934-020-00987-y

**Published:** 2020-08-17

**Authors:** David Eldred-Evans, Henry Tam, Andrew P. T. Smith, Mathias Winkler, Hashim U. Ahmed

**Affiliations:** 1 Imperial Prostate, Division of Surgery, Department of Surgery and Cancer, Faculty of Medicine, Imperial College London, London, SW7 2AZ UK; 2 Department of Urology, Imperial College Healthcare NHS Trust, London, UK; 3 Department of Radiology, Charing Cross, Imperial Healthcare NHS Trust, Fulham Palace Road, London, W6 8RF UK; 4 North West London Pathology, Charing Cross Hospital/Hammersmith Hospital, Imperial College Healthcare NHS Trust, London, W2 1NY UK

**Keywords:** Prostate cancer, Focal therapy, Magnetic resonance imaging, Multi-parametric MRI

## Abstract

**Purpose of Review:**

Rapid advances in imaging of the prostate have facilitated the development of focal therapy and provided a non-invasive method of estimating tumour volume. Focal therapy relies on an accurate estimate of tumour volume for patient selection and treatment planning so that the optimal energy dose can be delivered to the target area(s) of the prostate while minimising toxicity to surrounding structures. This review provides an overview of different imaging modalities which may be used to optimise tumour volume assessment and critically evaluates the published evidence for each modality.

**Recent Findings:**

Multi-parametric MRI (mp-MRI) has become the standard tool for patient selection and guiding focal therapy treatment. The current evidence suggests that mp-MRI may underestimate tumour volume, although there is a large variability in results. There remain significant methodological challenges associated with pathological processing and accurate co-registration of histopathological data with mp-MRI. Advances in different ultrasound modalities are showing promise but there has been limited research into tumour volume estimation. The role of PSMA PET/CT is still evolving and further investigation is needed to establish if this is a viable technique for prostate tumour volumetric assessment.

**Summary:**

mp-MRI provides the necessary tumour volume information required for selecting patients and guiding focal therapy treatment. The potential for underestimation of tumour volume should be taken into account and an additional margin applied to ensure adequate treatment coverage. At present, there are no other viable image-based alternatives although advances in new technologies may refine volume estimations in the future.

## Introduction

The aim of focal therapy is to retain equivalent oncological outcomes to whole-gland therapies while reducing the side effects associated with these treatments. Preservation of normal tissue is fundamental to the approach and this relies on an accurate assessment of tumour volume. An accurate knowledge of tumour volume allows maximal therapy to be directed to the target area while minimising damage to the surrounding structures such as neurovascular bundles, bladder neck and rectal wall.

In the early stages of focal therapy, volume assessment was achieved by transperineal template mapping biopsy (TPM) which was invasive and had related adverse events [1]. There was therefore a need for a simple, non-invasive and accurate method to assess tumour volume. In other solid-organ cancers, imaging is routinely used to assess volume prior to any organ-sparing surgery such as partial nephrectomy or partial mastectomy. The aim of this article is to review the role of tumour volume assessment for focal therapy planning and to critically evaluate the most recent published evidence for different imaging modalities to estimate tumour volume.

## Rationale for Tumour Volume Assessment

### Focal Therapy Treatment Planning

Focal therapy encompasses a wide range of approaches that allow selective ablation of target areas. This may be delivered by a variety of energy modalities including high-intensity focused ultrasound (HIFU), cryotherapy, photodynamic therapy, focal laser ablation, focal brachytherapy, irreversible electroporation and radiofrequency ablation as well as interstitial drug injections. The principles of focal therapy planning are similar across all these techniques and rely on a balance between:

1. Ensuring maximal safe energy delivery to area(s) of cancer with an appropriate margin
2. Minimising damage to normal prostatic tissue and adjacent anatomical structures.

This requires precise mapping and contouring of the treatment area dependent on the volume of the lesion. An underestimation of tumour volume may result in inadequate coverage of the target area leaving residual significant disease and poor long-term efficacy. An overestimation of tumour volume increases the risk of damage to normal prostate tissue and structures such as neurovascular bundles, bladder neck, external sphincter and rectum. The coverage area of focal treatments maybe be lesion-based, quadrant, hemi-ablation or sub-total. The degree to which the precision of tumour volume is important will depend on the treatment strategy chosen as well as the energy source with newer devices offering greater precision of tissue destruction.

### Risk Stratification: Clinically Insignificant Disease

The evidence for which men will benefit from active treatment of prostate cancer is evolving. It has been argued that low-grade and low-volume lesions do not have the typical hallmarks of cancer, certainly do not behave aggressively and may be regarded as clinically insignificant [2]. The ProtecT trial, which randomised men to active monitoring, surgery or radiotherapy in men diagnosed via PSA screening found no difference in prostate cancer-specific mortality at a median follow-up of 10 years [3]. The PIVOT and SPCG-4 RCTs show that the benefit of treatment resided in the high-risk group and possibly in intermediate-risk men too [4, 5]. There is a clear need for improved methods of risk stratifying men so that treatment can be directed towards those who are more likely to derive a cancer-specific mortality benefit.

The most widely used definition of clinically insignificant disease is based on the histopathological parameters set out by Stamey and Epstein [6]. Insignificant prostate cancer is defined on whole-mount prostatectomy as a tumour volume ranging from 0.2 to 0.5 cm^3^, no Gleason patterns 4 or 5 and organ confined. The original paper by Stamey et al. [6] described a single parameter of tumour volume ≥ 0.5 cm^3^ from a cystoprostatectomy series based on an 8% lifetime risk of being diagnosed with clinically significant cancer. Epstein et al. reported a volume threshold < 0.2 cm^3^ as being insignificant if the criteria of no capsular penetration were applied.

This tumour volume criterion has been generally considered too stringent and the definition of < 0.5 cm^3^ has been applied as the threshold for insignificant disease. In recent years, there has been a growing consensus that the 0.5 cm^3^ volume threshold remains too conservative. In a contemporary cystoprostectomy cohort applying the Stamey criteria, Winkler et al. [7] identified a higher threshold of 1.09 cm^3^. An analysis of the radical prostatectomy specimens from the European Randomized Study of Screening for Prostate Cancer found that grade and stage were the strongest determinants of lifetime risk estimates for prostate cancer [8]. When tumour volume was considered with organ confined Gleason 6 disease, a higher threshold of at least 1.3 ml for the index lesion and 2.5 ml for the total cancer volume was observed.

### Patient Selection

Tumour volume has a fundamental role in patient selection for focal therapy and the success of treatment depends on identifying men with the appropriate burden of disease. The ideal case for focal therapy is a small-volume intermediate grade prostate cancer which is localised on multi-parametric MRI (mp-MRI) [9]. A recent expert consensus panel explored the range of tumour volumes which would be acceptable for focal therapy. The consensus was reached that an index lesion with an mp-MRI-derived volume up to 1.5 ml was suitable for treatment. There was further agreement that this volume threshold could be increased to 3 ml provided the lesion was localised to one hemi-gland and the energy source was capable of ablating this volume with an acceptable margin.

There has been a shift in the consensus opinion towards focal therapy as a strategy for treatment of intermediate rather than low-risk disease. The expert panel confirmed that Gleason 3+4 disease represents the optimum grade for focal therapy although there was a lack a consensus for treatment of higher risk disease. This shift towards treatment of intermediate-risk disease is in line with widespread consensus that men who are low risk should undergo active surveillance and even focal therapy would be overtreatment in this group. Furthermore, the promising medium term outcomes of focal therapy which are emerging from prospective cohort registry studies also support this trend. The 5-year outcomes of HIFU have been reported from a UK registry analysis of 625 patients showing failure-free survival was 88% with a median follow-up of 56 months [10]. The functional outcomes have been summarised in recent meta-analysis comparing patient reported outcomes measures of different whole gland therapies with HIFU and active surveillance [11]. The follow-up was inevitably shorter in the HIFU trials but there was no significant deterioration of sexual function or incontinence at 1 year. This is consistent with the findings of Yap et al. which found that although potency deteriorated at 1 and 3 months post-HIFU, it returned to baseline by 6 months [12].

### The Need for an Alternative Volume Estimate

Prior to the emergence of focal therapy, there was a lack of an accurate and robust method of measuring pre-operative tumour volume. Previous attempts to determine tumour volume based on PSA level or digital rectal examination found that neither reliably correlated with volume on radical prostatectomy [13, 14]. The clinical Epstein criteria include indirect measurements of volume by number of positive cores and percentage of cancer on TRUS biopsy. Due to the sampling error inherent with this biopsy approach, these parameters have not been consistently shown to correlate with tumour volume on radical prostatectomy and have been calibrated to ensure significant disease is not missed rather than calibrated to prevent overtreatment [15–18].

Transperineal template mapping biopsy (TPM) has been shown to provide an accurate pre-operative risk assessment based on tumour volume definitions of clinically significant disease [19]. A cancer core length of ≥ 6 mm has been shown to predict lesions ≥ 0.5 ml in volume through a process of simulation against a radical prostatectomy cohort [19]. During the early stages of focal therapy, TPM was seen as an essential tool in selecting and risk stratifying men prior to treatment [20]. However, the high sampling density and requirement for a general anaesthetic placed a significant burden on both the patient and healthcare system [1].

The current biopsy pathway is being re-defined with a shift towards MRI-targeted biopsy. 3D histopathological models reconstructed from TPM biopsies were used to show that a single biopsy needle targeted to the maximum lesion diameter on mp-MRI leads to the correct Gleason grade in nearly all cases [21]. There is evidence from recent randomised controlled trials which confirm that a pre-biopsy MRI with or without targeted biopsy is superior to standard TRUS biopsy [22]. Although MRI-US-targeted biopsy may enhanced detection of clinically significant disease, the effect on estimation of tumour volume remains under investigation.

The studies attempting to re-calibrate maximum core length on targeted biopsy and tumour volume on radical prostatectomy have reported variable results [23, 24]. Baco et al. [23] found a weak correlation between maximum core length on elastic MR-TRUS image fusion and tumour volume on radical prostatectomy (*r* = 0.466). In this study, mp-MRI was a more accurate predictor of tumour volume (*r* = 0.663) than maximum cancer core length on targeted biopsy. Both cognitive and software-based targeted biopsy have a known targeting error which may be due to registration error [25], patient movement, prostate deformation or needle placement errors [26]. In comparison with TPM, this variability places an increasing importance on imaging to accurately detect and estimate tumour volume prior to focal therapy.

## Multi-parametric MRI

The development of focal therapy has been facilitated by advances in imaging technologies. mp-MRI has become the standard imaging modality to detect and localise the index lesion. It provides an evaluation of the whole prostate in contrast to biopsy which samples only a very small proportion of the gland. There is extensive evidence that mp-MRI can reliably identify clinically significant disease ≥ 0.5 cm^3^ in volume with a high sensitivity and negative predictive value [27].

However, a volume measurement requires a higher level of spatial recognition than localisation or detection. There are an increasing number of studies evaluating volumetric assessment of lesions against radical prostatectomy specimens as the reference standard. Table 1 provides a summary of the studies including the differences in MRI techniques, contouring procedures and registration methods. There is significant heterogeneity across the literature with both underestimation and overestimation for tumour volume reported. There is also a high standard deviation within studies where tumour volume errors can range from − 136 to + 178%.

**Table 1:** Studies evaluating volumetric assessment of lesions against radical prostatectomy specimens as the reference standard

Study	Patients (*n*)	Technical details of MRI				MRI volume calculation		Radical prostatectomy specimen			
		Field strength	Coil	Sequences	Image slice thickness	Volume measured	Scoring system	Method of volume calculation	Volume	Tissue shrinkage correction factor	Slice thickness
Nakashima et al. 2004 [28]	95	1.5 T	PPA + ERC	T2W, DCE	5 mm	T2W	No scoring system	Maximal diameter	Manual plainmetry	1.1	NR
Mazaheri et al. 2009 [29]	42	1.5 T	ERC + PPA	T2W, DWI	3 mm	T2W + ADC map	No scoring system	Manual plainmetry	Calibration ruler	None	3–5 mm
Lemaitre et al. 2009 [30]	27	1.5 T	PPA	DCE	4 mm	DCE	No scoring system	Semi-automated software	-	1.5	3 mm
Turkbey et al. 2012 [31]	135	3 T	ERC	T2W, DWI, DCE	3 mm	All sequences	Number of positive sequences	Software	Maximum tumour diameter	1.15	4 mm
Isebaert et al. 2012 [32]	75	1.5 T	PPA	T2W, DWI, DCE	3 mm	NR	No scoring system	MeVisLab software	Manual	1.33	3 mm
Anwar et al. 2014 [33]	20	3 T	PPA + ERC	T2W	5 mm	T2W	No scoring system	Matlab software	Spherical volume formula	Elastic registration	3 mm
Engelhard et al. 2014 [34]	55	1.5 T	PPA + ERC	T2W, DWI, DCE	3 mm	All sequences	0–4 scoring system	NR	Visual estimation	None	4–5 mm
Cornud et al. 2014 [35]	84	1.5 T	ERC	T2W. DWI, DCE	3.5 mm	All sequences	No scoring system	Plainmetry	Software (Hamamatsu)	None	4 mm
Rud et al. 2014 [36]	199	1.5 T	ERC	T2W, DWI	NR	T2W	No scoring system	Ellipsoid formula	Manual plainmetry	1.15	4–5 sections per gland
Bratan et al. 2014 [37]	202	1.5 T (71 patients) 3 T (131 patients)	PPA	T2W, DWI, DCE	3 mm	All sequences	Likert	Software (OsiriX)	Software (Matlab)	None	3 mm
Le Nobin et al. 2014 [38]	37	3 T	PPA	TW2, DWI, DCE	3 mm	T2W + ADC map	Likert	Co-registration software FireVoxel		Elastic registration	5 mm
Baco et al. 2015 [23]	135	1.5 T	PPA	TW2, DWI, DCE	NR	NR	PI-RADS	Ellipsoid formula	Simplified 3D estimation	None	3–5 mm
Radtke et al. 2016 [39]	120	3 T	PPA	TW2, DWI, DCE	3 mm	NR	PI-RADS	Medical Imaging Toolkit software	NR	1.5	NR
Martorana et al. 2016 [40]	157	1.5 T	ERC + PPA	TW2, DWI,DCE	3 mm	T2W	PI-RADS v2	Biopsee software	Elipsiod formula	None	NR
Priester et al. 2017 [41]	114	3 T	PPA + ERC	TW2, DWI, DCE	1.5 mm	T2W	Likert	ProFuse software	3D mold	Elastic registration	4.5 mm

*PPA*, pelvic phased-array coil; *ERC*, endorectal coil; *MRSI*, MR spectroscopy imaging; *NR*, not reported

### Evidence for Overestimation

There has been a rapid progression in technology and experience with mp-MRI over the last decade. The earlier studies were more likely to conclude that mp-MRI overestimated tumour volume but did not include software-based registration and relied on lower magnetic field strengths, including 0.5 T [42], non-multi-parametric sequences [30, 42]. Overestimation may also be attributable to methodological differences in sectioning the prostate gland and applying a correction factor for tissue shrinkage which varied from 1.14 to 1.5 [43, 44].

The decision regarding the degree of correction factor has a significant impact on the balance between overestimation and underestimation. Turkbey et al. [31] reported an identical analysis with and without a shrinkage factor correction. Without a shrinkage factor correction, mp-MRI overestimated tumour volume by 7% but after shrinkage factor correction, there was an underestimation of 7%. There is evidence that tissue shrinkage is not uniform between specimens and varies depending on fixation and mounting methods [45]. Recent studies have developed novel elastic 3D co-registration software to limit this bias and allow an individualised correction factor for each specimen [38].

### Evidence for Underestimation

The studies conducted in the modern mp-MRI era generally describe that mp-MRI underestimates tumour volume, although there remains wide variability in the degree which ranges from 4 to 97% [40]. This underestimation occurs irrespective of MRI suspicion score or tumour characteristics[41] but may be biased by further challenges associated with preparation of the radical prostatectomy specimen.

Preparation of the histopathological slides leads to differences in angles, shape and depth compared with the MRI images. The sectioning plane of prostatectomy specimens may not reflect the MRI plane and the prostate shape can be altered by histopathological tissue processing. To improve the accuracy of registration, studies have used 3D patient-specific molds which improve the alignment of the specimens to the MRI images [41, 46]. However, these to not resolve all the registration challenges as while MRI slice thickness has improved to 1.5 mm, the majority of radical prostatectomy specimens underwent 3- to 5-mm step-sectioning (Table 1). The lack of a 1:1 slice correlation creates a systematic bias in the apex to base axis although the direction of effect depends on the exact method for calculating volume which is not reported in most studies. A study which stratified the level of underestimation by their axis reported that mp-MRI volumes were least accurate in the apex-base plane and most accurate in the axial plane with a MRI to histology slice ratio of 1.5:4 mm [41].

Histologically, it is speculated that underestimation may be attributable to the characteristic histological features at the boundary of the index lesion. Langer et al. have previously described histologically sparse areas of prostate cancer containing normal tissue intermixed with malignant epithelium which may not be visible on both T2WI and ADC maps [47]. These sparse regions may occur at the periphery of the index lesion meaning that mp-MRI will inherently underestimate the boundaries of visible lesions.

There is contradictory data on the effect of the MRI suspicion score, Gleason pattern and tumour volume on the degree of underestimation. The largest current study by Bratan et al. prospectively evaluated 202 radical prostatectomies and analysed the MRI and tumour characteristics which enhanced accuracy of mp-MRI-derived volume. The multi-variate analysis showed that Likert scores 4–5, Gleason score ≥ 7 and volume ≥2 ml had a more accurate mp-MRI volume estimation. Given that these large high-grade cancers are also easier to detect on mp-MRI [48], it might be expected that this would translate into a more accurate volume estimation.

However, using validated 3D co-registration software, Le Nobin et al. have reported the unexpected finding that larger tumours (> 1 ml) with a higher MRI or Gleason score had a more pronounced volume underestimation [38]. The authors suggest that this may be related to the more solid histological components of high-grade cancers meaning they manifest as clear dark areas on the ADC map. Given that PI-RADS v2 (Prostate Imaging-Reporting and Data System) recommends peripheral zone lesions are measured on ADC, it is possible that the radiologist’s attention is directed towards the darker areas which inherently excludes the less conspicuous surrounding non-solid lower-grade regions for the volume estimation.

### Optimising MRI Volume Measurements

#### Optimal Method of Measurement

The PI-RADS v2.1 (Prostate Imaging-Reporting and Data System) guidelines provide the minimal requirements for measurement of volume which are to report a single measurement of a suspicious lesion on an axial image unless it is not clearly delineated, in which case the measurement should be on the image which best depicts the finding [49]. If the largest dimension of the lesion is on sagittal or coronal images, this measurement should also be reported.

The maximal diameter is a simple measurement which is feasible to obtain in clinical practice. It has been used as an inexpensive surrogate for tumour volume in radical prostatectomy specimens following studies comparing different methods for tumour size estimation [50]. The role of maximum diameter for mp-MRI-derived volume estimation is less well-established. Nakashima et al. [28] found that the maximal tumour diameters on MRI and radical prostatectomy specimens should be limited to tumours larger than 1.0 cm in diameter.

Alternative methods include three-dimensional quantification based on an ellipsoid formula or plainmetry. Planimetric volume measurement is presumed to be the most accurate technique and the majority of studies evaluating mp-MRI-derived tumour volumes adopt this approach. Plainmetry requires contouring of the lesion on each axial slice and places a significant additional time burden on the reporting radiologist.

At present, there remains no agreed method of measuring tumour volume on mp-MRI. Plainmetry is not routinely used in clinical practice although there is potential for this to change with further research into semi-automated or fully automated volumetric measurement software [51]. This issue was discussed in a recent expert consensus meeting and the panel concluded that there was not sufficient evidence to recommend any optimal method for measuring tumour volume on mp-MRI [52].

#### Optimal mp-MRI Sequence

**Fig. 1:**
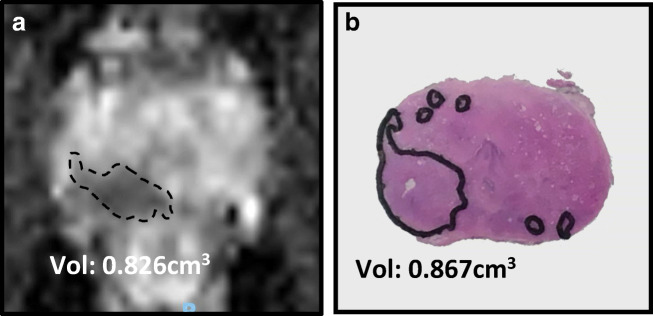
mp-MRI lesion volume compared with pathology volume

The early methods of estimating tumour volume were based on unenhanced T2W imaging alone. The addition of functional MRI sequences, such as diffusion-weighted imaging (DWI) and dynamic contrast-enhanced (DCE), improved the sensitivity and specificity for detection of clinically significant disease [48]. The effect on tumour volume assessment has been investigated in a limited number of studies [29, 32, 35] which find that T2W alone has a poor correlation with pathological volume (*r* = 0.21) [35]. DWI and ADC maps are consistently reported as the most accurate sequences with a high correlation coefficient (*r* = 0.75) [32]. Figure 1 shows an mp-MRI with an accurate tumour volume estimated on ADC map. DCE was the lowest performing sequence which is likely secondary to the lower spatial resolution of this sequence [29, 35].

An alternative approach is a measurement based on a combination of sequences acknowledging that all sequences underestimate tumour volume. Two studies have concluded that more accurate volume can be determined by the largest volume from any individual sequence [35, 45]. However, this approach leads to an overestimation of tumour volume by 16% [35]. Overall, the current evidence supports the use of DWI and ADC maps as the most accurate sequence for assessing tumour volume in the peripheral zone. These findings are reflected in PIRADS v2.1 which recommends that ADC should be used for assessing tumour volume in the peripheral zone [49]. T2W is recommended for the transition zone reflecting a lack of evidence for an optimal sequence for lesions in this region.

A potential benefit of ADC maps is that they allow a more objective assessment of volume as the values are proportional to the diffusion and perfusion characteristics of the tissue. There has been significant interest in establishing an objective ADC threshold to provide an automated method of assessing tumour volume. A voxelwise analysis by Mazaheri et al. [29] found that ADC cutoff values of 0.0014 and 0.0016 mm^2^/s improved the accuracy of volume measurements. The challenge for these models is that there is a large spread of ADC values inside the MRI lesion which can mean parts may not be automatically detected or there could be overdiagnosis depending on the threshold [53].

#### Other Factors

The zonal anatomical location of the index lesion could have an impact on accuracy of volume estimation. There are a few studies which categorise peripheral and transition zone lesions [36, 38, 41]. These suggest that transition zone lesions may be associated with more variability in tumour volume estimation [36] although peripheral zone lesions account for the majority in the analysis. The heterogeneous appearance of the transition zone makes it more challenging to define accurate boundaries in comparison with the peripheral zone lesions.

There are a wide range of other MRI technical characteristics which have been investigated in the existing literature which seem to have a limited effect on volume assessment. The influence of field strength was tested in a multi-variate analysis by Bratan et al. [37]. Despite the higher spatial resolution of 3 T, there was no significant difference in accuracy between 1.5 and 3 T. The impact of an endorectal coil has also been debated as it deforms the peripheral zone and may modify tumour contours in this region. Studies have been completed at 3 T and 1.5 T both with and without an endorectal coil. There is minimal evidence that the presence of an endorectal coil has a significant impact on tumour volume accuracy.

### Optimising Focal Therapy Treatment Margins

To compensate for the underestimation of mp-MRI tumour volumes, there have been attempts to estimate an appropriate treatment margin to ensure full coverage of the index lesion. The 2015 Focal Therapy Consensus meeting recommended a circumferential margin of 5 mm around a lesion accounting for a 2–3 mm known registration error [25] and the underestimation by mp-MRI [54].

The principle of an adequate surgical margin surrounding a tumour is standard for all organ-conserving surgery. There has been extensive research into the appropriate margins for operations such as partial nephrectomy, partial penectomy and partial ureterectomy. For focal therapy, there are challenges to overcome due to the absence of a post-operative specimen to evaluate margin status. This has presented challenges for research into margin status and studies rely on extrapolation from radical prostatectomy specimens which have an inherent selection bias as well as the problems of accurate co-registration between histology and MRI.

Cornud et al. [35] recommended a ‘target volume’ calculated on the largest tumour area on each axial slice from any sequence, but this resulted in an overestimation of pathological tumour volume by 44%. Recent work has attempted to quantify this into an exact margin using a simulated cylindrical treatment volume or the widest margin to achieve complete histological tumour distribution in all patients. These different methodologies have produced variable results with margins ranging from 5 mm [33] up to 13.5 mm [41, 55].

This variation highlights the need for individualised treatment margins which are determined based on the appropriate therapeutic risk-benefit ratio for each patient. The optimal margin is influenced by multiple patient-specific and operative factors. Tumour volume is an important component for the surgeon to consider along with other interrelated variables such as index lesion location, histological characteristics and energy modality.

## Other Imaging Modalities

### Transrectal Ultrasound

Transrectal ultrasound (TRUS) was the first major development in prostate cancer imaging. It revolutionised prostate cancer diagnostics by allowing the boundaries of the prostate to be visualised and providing the foundation for systematic biopsy [56]. Compared with other imaging modalities, TRUS is a fast, cost-effective and portable procedure which provides good soft-tissue contrast without the need for ionising radiation or administration of contrast agents. However, b-mode TRUS has demonstrated limited sensitivity and specificity for detecting and localising prostate cancer [57]. Given the lack of diagnostic accuracy, it is predictable that studies using b-mode TRUS have shown it is a poor predictor of tumour volume[13, 14].

Ultrasound is undergoing rapid technological advancements. Developments in high-resolution ultrasound operating up to 29 Hz allow superior spatial resolution over traditional b-mode imaging [58]. This may allow improved diagnostic accuracy and tumour volume assessment in the peripheral zone although the reduced penetration depth may limit assessment of the anterior gland. This has been combined with 3D scanning techniques to monitor the longitudinal growth of tumour volume in an orthotopic mouse model [59]. The 3D rendering showed good correlation for prostate tumour volume measurements performed in vivo with autopsy (*r* = 0.95).

Similar to the development of mp-MRI, there has been interest in combining US modalities into a ‘multi-parametric ultrasound’. If a combination of anatomical and functional parameters can be shown to accurately detect and localise the index lesion, this will be a significant step forward for focal therapy treatment planning due to the real-time monitoring available through ultrasound. The early results for multi-parametric ultrasound are encouraging [60] and there are ongoing randomised controlled trials which will provide a robust comparison with mp-MRI [61]. At present, there is limited data on the performance of these modalities for determining tumour volume while we await further evidence on diagnostic accuracy.

### CT

Although computed tomography (CT) is a widely used modality for the detection and localisation in many malignancies, it has a limited role in prostate cancer localisation or focal therapy planning and due to the inherent lack of soft tissue, contrast resolution so cannot reliably visualise prostate zonal anatomy or estimate volume of tumours. The prostate will generally appear on an unenhanced CT as a homogenous soft tissue structure, and prostate cancer will not be visualised unless gross extension is present [62].

### PET/CT

Although the role of choline PET/CT as a staging investigation is well established, the spatial resolution is limited to around 5 mm so its ability to accurately localise prostate cancer and estimate tumour volume has been debated. The role of choline PET/CT for focal therapy planning has not been investigated but it has been evaluated for delineation of the dominant intra-prostatic lesion for intensity modulated radiation therapy (IMRT) focal dose escalation. This requires a similar process of contouring the lesion as focal therapy planning and at present, mp-MRI is the standard technique.

The results for choline-based markers suggest they have a limited role for evaluation of tumour volume. Multiple studies have shown a poor correlation with tumour volume (*r* = 0.3) [63] and Bundschuh et al. found that the choline uptake pattern was inconsistent and no suitable threshold could be identified to fit histological volume [64]. Instead PSMA ligands such as 68Ga-labeled HBED-CC-PSMA or 18F-labeled DCFPyl are emerging as a promising alternative and appear to be more sensitive for detection of local and metastatic disease. The majority of studies focus on the detection of recurrent or metastatic disease. Preliminary studies evaluating PSMA/PET for IMRT focal dose have found that PET-derived volumes are significantly larger in some patients compared with MRI or prostatectomy volumes [65, 66] and that further correlation studies with co-registration of histopathological data are required.

## Conclusion

An accurate estimation of tumour volume is essential for focal therapy treatment planning. If the tumour volume is overestimated, the risk of complications increases, while an underestimation reduces the chance of effective cancer control. The advances in mp-MRI have provided a non-invasive method of assessing tumour volume although this may be underestimated by all sequences. At present, there are no other viable image-based alternatives for assessing tumour volume.

There is considerable variability across the literature in the results for all studies which likely reflects the variability in imaging techniques and methods of co-registration between studies. For further research to progress in this area, there needs to be a robust method for co-registration of histopathological data with imaging.
